# Engineering Resistance to Bacterial Blight and Bacterial Leaf Streak in Rice

**DOI:** 10.1186/s12284-021-00482-z

**Published:** 2021-04-23

**Authors:** Zhe Ni, Yongqiang Cao, Xia Jin, Zhuomin Fu, Jianyuan Li, Xiuyu Mo, Yongqiang He, Jiliang Tang, Sheng Huang

**Affiliations:** 1 State Key Laboratory for Conservation and Utilization of Subtropical Agro-bioresources, College of Life Science and Technology, Guangxi University, 100 Daxue Road, Nanning, 530004 Guangxi China; 2 Present address: College of Basic Medical Sciences, Guangxi Medical University, 22 Shuangyong Road, Nanning, 530021 Guangxi China; 3 College of Agriculture, Guangxi University, 100 Daxue Road, Nanning, 530004 Guangxi China

**Keywords:** *Xanthomonas oryzae*, Transcription activator-like effectors, Susceptibility gene, Disease resistance, Genome editing rice

## Abstract

**Background:**

*Xanthomonas oryzae* (*Xo*) is one of the important pathogenic bacterial groups affecting rice production. Its pathovars *Xanthomonas oryzae* pv. *oryzae* (*Xoo*) and *Xanthomonas oryzae* pv. *oryzicola* (*Xoc*) cause bacterial blight and bacterial leaf streak in rice, respectively. *Xo* infects host plants by relying mainly on its transcription activator-like effectors (TALEs) that bind to host DNA targets, named effector binding elements (EBEs), and induce the expression of downstream major susceptibility genes. Blocking TALE binding to EBE could increase rice resistance to the corresponding *Xo*.

**Findings:**

We used CRISPR/Cas9 to edit the EBEs of three major susceptibility genes (*OsSWEET11*, *OsSWEET14* and *OsSULTR3;6*) in the rice varieties Guihong 1 and Zhonghua 11. Both varieties have a natural one-base mutation in the EBE of another major susceptibility gene (*OsSWEET13*) which is not induced by the corresponding TALE. Two rice lines GT0105 (from Guihong 1) and ZT0918 (from Zhonghua 11) with target mutations and transgene-free were obtained and showed significantly enhanced resistance to the tested strains of *Xoo* and *Xoc*. Furthermore, under simulated field conditions, the morphology and other agronomic traits of GT0105 and ZT0918 were basically the same as those of the wild types.

**Conclusions:**

In this study, we first reported that the engineering rice lines obtained by editing the promoters of susceptibility genes are resistant to *Xoo* and *Xoc*, and their original agronomic traits are not affected.

**Supplementary Information:**

The online version contains supplementary material available at 10.1186/s12284-021-00482-z.

## Findings

Rice is an important staple crop in the world and its yields are easily influenced by many factors, of which the pathogens *Xanthomonas oryzae* pv. *oryzae* (*Xoo*) and *Xanthomonas oryzae* pv. *oryzicola* (*Xoc*) cause bacterial blight and bacterial leaf streak, respectively (Nino-Liu et al., 2006). *Xoo* and *Xoc* have a special class of type III effectors, called transcription activator-like effectors (TALEs), which play important roles in pathogenicity. Typical TALEs consist of three domains: the N-terminal domain that contains the type III secretion signal, the C-terminal domain that plays a role in nuclear localization and transcriptional activation, and the central highly conserved repeat units. Each repeat unit contains 33–35 amino acids, in which the 12th and 13th amino acids are variable, called the repeat variable di-residue (RVD). Different RVDs can recognize different bases of target DNA, and the TALE containing multiple RVDs can recognize a specific DNA sequence (Mak et al., 2012). The main function of a TALE is binding to the effector binding element (EBE) in the host genome to induce downstream gene expression (Li et al., 2012). *Xoo* and *Xoc* cause rice diseases by relying mainly on their TALEs, which bind to host EBEs and induce the expression of downstream major susceptibility genes (Kay and Bonas, 2009; Moscou and Bogdanove, 2009). The genes of *OsSWEET* family in rice are important susceptibility genes induced by *Xoo* TALEs. There are more than 20 *OsSWEET* genes in rice, but in nature, *Xoo* can induce only three of them: *OsSWEET11*, *OsSWEET13* and *OsSWEET14* (Chen et al., 2010; Streubel et al., 2013; White et al., 2009; Yang et al., 2006). The TALEs capable of inducing *OsSWEET11* and *OsSWEET13* include PthXo1 and PthXo2, respectively, and there are several TALEs that can induce *OsSWEET14*, including PthXo3, AvrXa7, TalC and TalF (Antony et al., 2010; Chu et al., 2006; Hutin et al., 2015; Tran et al., 2018; Yang et al., 2006). The four *OsSWEET14*-inducing TALEs recognize different EBEs. Two of them (PthXo3 and AvrXa7) are only present in the strains from Asia while the others (TalC and TalF) are present in the strains from Africa (Antony et al., 2010; Streubel et al., 2013; Yu et al., 2011; Oliva et al., 2019). In addition, it has been demonstrated that the TALE Tal2g is the major virulence factor of *Xoc*, by which the susceptibility gene *OsSULTR3;6* in rice is induced for full disease development (Cernadas et al., 2014). Genome data (the NCBI database) mining showed that *Tal2g*-encoding gene is present in all of the *Xoc* strains except YM15 which is without TALEs. At present, the prevention and control of bacterial blight and bacterial leaf streak mainly depends on farm chemicals, which are costly and environmentally unfriendly. Engineering disease-resistant rice by editing the EBEs of the susceptibility genes could be an alternative to prevent the diseases. The advent of CRISPR technology has increased the effectiveness of gene-editing in rice. To date, several rice varieties resistant to *Xoo* have been obtained by editing the susceptibility genes (Li et al., 2020; Oliva et al., 2019; Xu et al., 2019). However, no rice variety resistant to *Xoc* has been generated by gene editing. Here we demonstrate that editing the EBEs of the *Xoc* susceptibility gene *OsSULTR3;6* as well as the *Xoo* susceptibility genes *OsSWEET11* and *OsSWEET14* in rice leads to significant resistance to *Xoo* and *Xoc*.

**Fig. 1:**
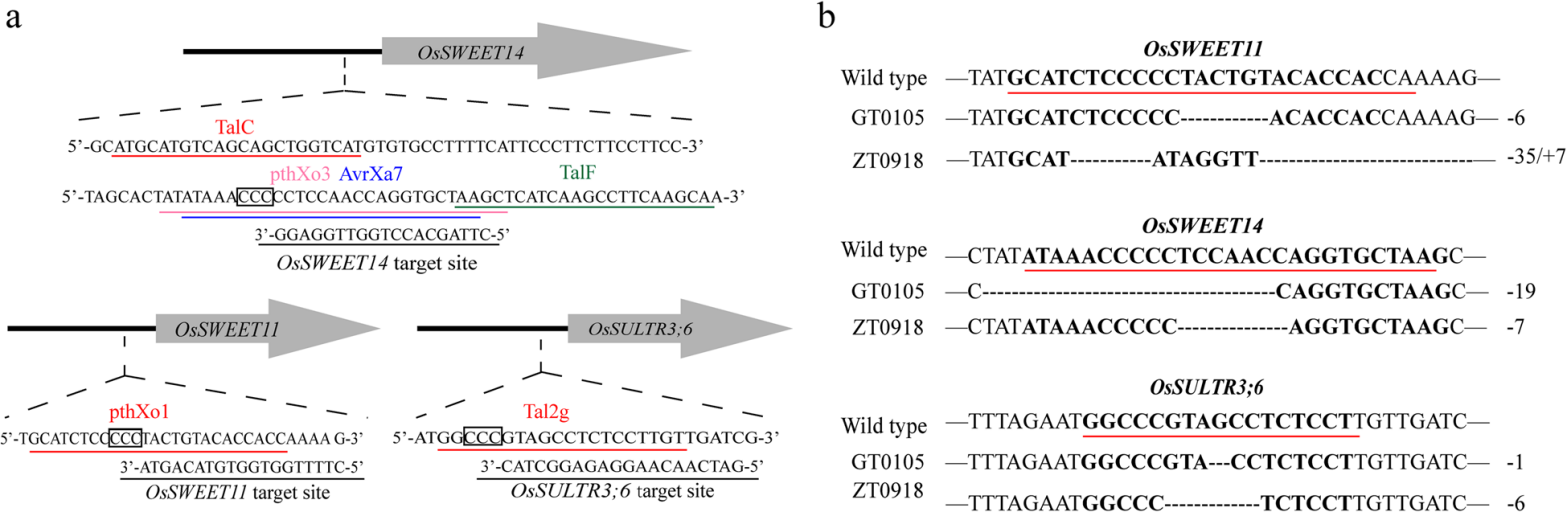
CRISPR-mediated editing of the susceptibility genes’ EBEs in rice. **a,** Schematic presentation of the target sites. The underlined sequences are the target sites. The sequences with black boxes represent the protospacer adjacent motif (PAM). **b,** Genotypes of the EBEs. Wild type represents the genotypes of Guihong 1 and Zhonghua 11. The sequences underlined in red are TALE binding sites. Deletions are indicated as dashes; the sign −/+ stands for deletion/ insertion; the number represents the number of deletion/insertion bases. The genotypes of *OsSWEET11*’s EBE in ZT0918 is shown in Supplemental Fig. 2

At first, we started with two rice varieties, Guihong 1 and Zhonghua 11, in both of which a natural one-base mutation was found in the EBE upstream of *OsSWEET13*; this mutation blocks the binding of the corresponding TALE PthXo2 to the EBE and the induction of *OsSWEET13* expression. The designed sgRNAs (Fig. 1a) targeting the EBE of *OsSWEET11* and the EBEs recognized by the Asia strains’ TALEs PthXo3 and AvrXa7 in *OsSWEET14* were respectively transformed into Guihong 1 and Zhonghua 11 to obtain EBE mutations and transgene-free rice lines by self-breeding. Afterward, an sgRNA (Fig. 1a) targeting the EBE of *OsSULTR3;6* was introduced into the edited Guihong 1 and Zhonghua 11 plants to obtain mutations in the EBE of *OsSULTR3;6* and transgene-free rice lines by self-breeding. Two rice lines named GT0105 (from Guihong 1) and ZT0918 (from Zhonghua 11) with target mutations and transgene-free were obtained. To verify that the edited lines were free from transgene, four pairs of primers specific to different regions of pYLCRISPR plasmid were designed and used to detect the genomes of the edited rice lines by PCR. The results showed that none of the primers could give any amplification product when using the total DNA of the edited lines as template (Supplemental Fig. 1), demonstrating that the plasmid used for genome editing had been removed in the edited rice lines obtained. All of the three target sites in GT0105 and ZT0918 were mutated, as revealed via PCR and sequencing (Fig. 1b and Supplemental Fig. 2). To detect potential off-target effects, we employed the CRISPR-P (Lei et al., 2014) and the CRISPR-GE (Xie et al., 2017) web tools to predict potential off-target sites relative to the edited targets in rice. Altogether, 46 putative off-target sites (10 for *OsSWEET11*, 15 for *OsSWEET14*, and 21 for *OsSULTR3;6*) which contain a PAM sequence and show high sequence similarity to the designed target sites (Supplemental Table 3) were found. We amplified all these putative off-target sites in the obtained edited rice lines by PCR and analyzed the PCR products by sequencing. The results showed that only single-nucleotide polymorphism (SNP) but no other mutation was found in 3 putative off-target sites (Supplemental Table 4), suggesting that no off-target mutation was introduced in the edited lines. Furthermore, we used the PlantCARE (Lescot et al., 2002) to determine whether the SNPs in the putative off-target sites affect any potential regulatory elements. No potential cis-acting regulatory element was found in the DNA regions containing the putative off-target sites, suggesting that the SNPs in the putative off-target sites may not have a regulatory effect.

**Fig. 2:**
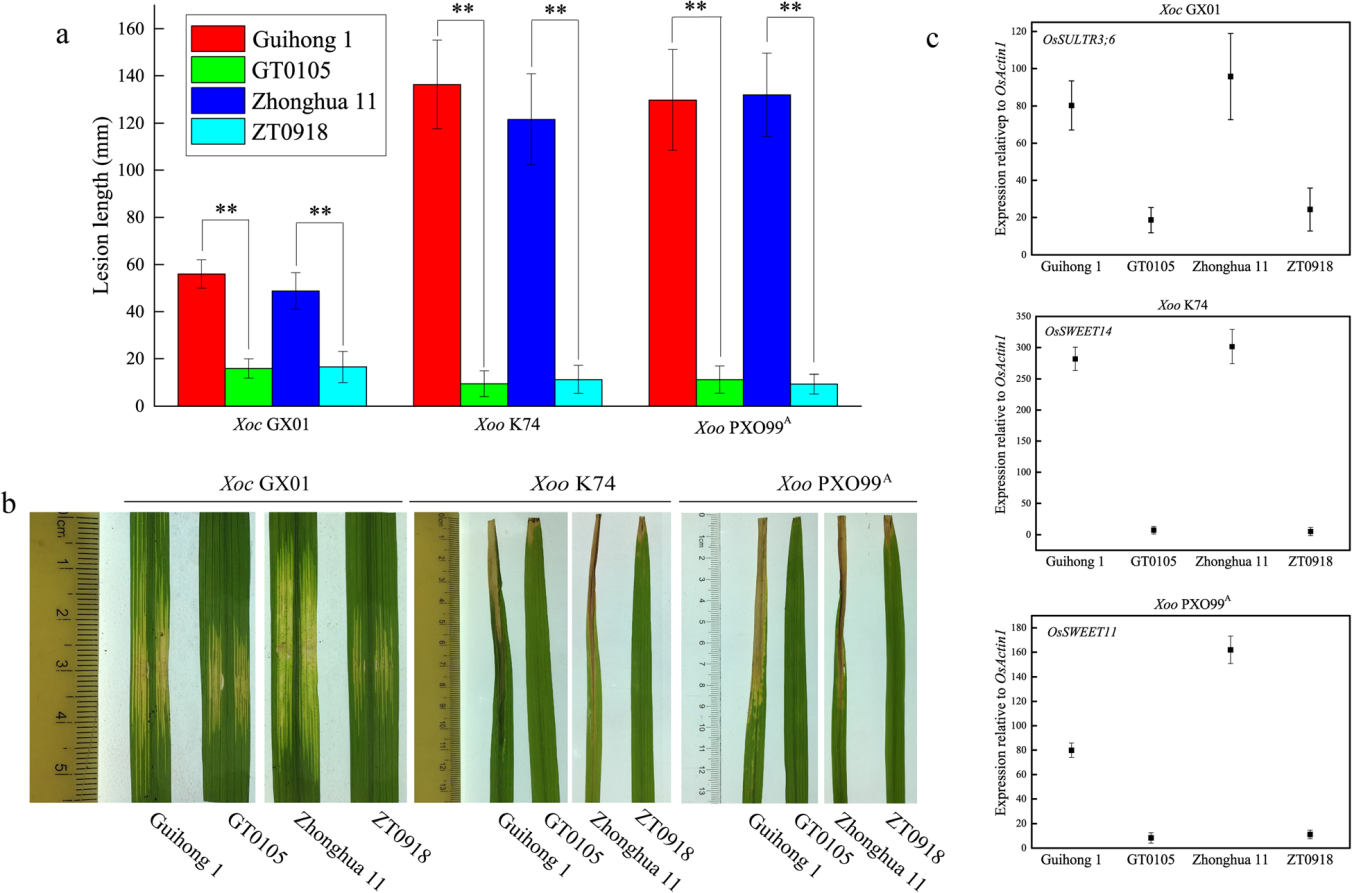
Detection of disease resistance and qRT-PCR analysis of susceptibility genes in disease-resistant rice lines. **a,** Length analysis of disease lesions. The bars indicate the standard deviations; ** indicates a significant difference between the lesion lengths via the *t*-test (*P* ≤ 0.01). **b,** Phenotypes of disease reactions in wild type rice varieties (Guihong 1 and Zhonghua 11) and edited rice lines (GT0105 and ZT0918) after inoculation with *Xoc* GX01, *Xoo* PXO99^A^ and *Xoo* K74. **c,** Relative expression levels of susceptibility genes in rice infected with *Xoc* GX01, *Xoo* K74 and *Xoo* PXO99^A^; the levels were determined via qRT-PCR. *OsActin1* was used as an internal standardized control

To test the resistance of the obtained genome-edited rice mutants to *Xoo* and *Xoc*, two *Xoo* strains (PXO99^A^ and K74) and one *Xoc* strain (GX01) were used for plant tests (Supplemental Table 1). As shown in Fig. 2a and b, the lesion length formed in the GT0105 leaves inoculated with GX01, K74 and PXO99^A^ was decreased by 71.54%, 93.07% and 91.36%, respectively, compared to the lesion length in the wild type rice leaves. Similarly, the lesion length in the ZT0918 leaves inoculated with GX01, K74 and PXO99^A^ was reduced by 66.0%, 90.69% and 92.92%, respectively. These indicate that the resistance of GT0105 and ZT0918 to *Xoo* and *Xoc* was significantly improved compared with that of their wild type. Further detection of the expression of disease susceptibility genes targeted by the cognate TALEs was performed by qRT-PCR. The result showed that under infection with different strains, the expression of the susceptibility genes in GT0105 and ZT0918 was significantly reduced compared to the wild type rice (Fig. 2c). This indicates that after editing, the promoters of the susceptibility genes can escape the corresponding TALE induction, leading to resistance to *Xoo* and *Xoc*.

**Fig. 3:**
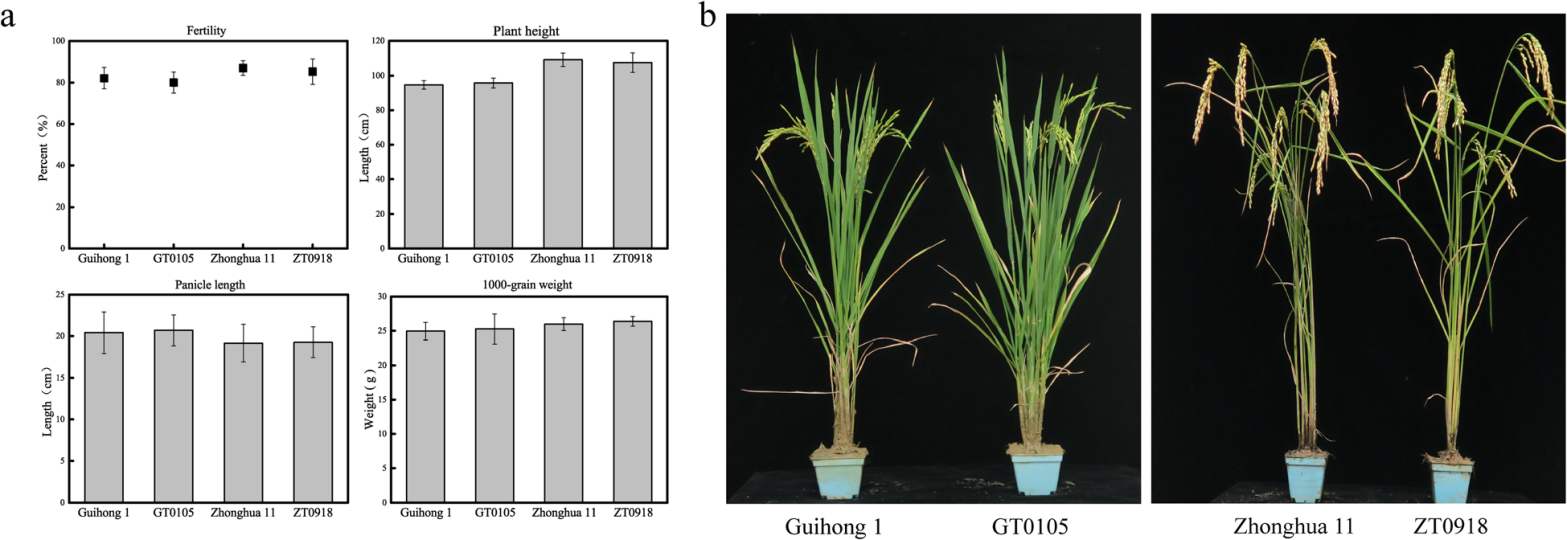
Agronomic traits in disease-resistant rice lines as compared to the parental controls. **a**, Agronomic trait (fertility, panicle length, plant height and 1000-grain weight) analysis of rice lines. **b**, Morphology of the assessed rice lines

To determine whether the agronomic traits of the edited rice lines were affected by the genome-editing, we simulated field conditions of rice cultivation to determine the agronomic characteristics of the edited rice lines. In our experiments, we found that GT0105 and ZT0918 were basically the same as the wild-type rice with respect to fertility, panicle length, plant height and 1000-grain weight (Fig. 3a). As shown in Fig. 3b, no significant morphological difference was observed between the genome-edited rice and the wild-type rice, indicating that editing the EBEs of the disease susceptibility genes did not affect their original biological functions. These data demonstrate that under similar conditions, GT0105 and ZT0918 performed similarly to wild-type parents in terms of basic agronomic characteristics.

Here we first report that the engineer rice lines obtained by editing the promoters of susceptibility genes are endowed with resistance to *Xoo* and *Xoc* without affecting other agronomic traits. As mentioned above, three *Xoo*-susceptable genes (*OsSWEET11*, *OsSWEET13* and *OsSWEET14*) have been identified so far. In this work we only edited *OsSWEET11* and *OsSWEET14* in consideration of the existence of a natural one-base mutation within the *OsSWEET13* EBE in the parent rice varieties. However, recent studies have shown that *Xoo* strains from different regions contain different PthXo2 TALEs, which have a variation in their RVDs and some of them can recognize the *OsSWEET13* EBE with a single-base mutation (Oliva et al., 2019; Xu et al., 2019). In addition, as described above, there are four EBEs in *OsSWEET14*, which are recognized by different TALEs, respectively. This work only edited the EBEs recognized by the TALEs (PthXo3 and AvrXa7) present in *Xoo* Asian isolates but not the EBEs recognized by the TALEs (TalC and TalF) present in African isolates. Therefore, the obtained edited rice lines might not be resistant to the *Xoo* strains with either variant PthXo2 or TalC/TalF. To overcome these shortcomings, we will edit the rest EBEs of *OsSWEET14* as well as the EBE of *OsSWEET13* in the obtained edited varieties GT0105 and ZT0918 to prevent the *OsSWEET* genes being induced by TalC, TalF and variant PthXo2 and generate rice varieties with broad-spectrum resistance to bacterial blight and bacterial leaf streak.

In summary, two rice lines with significantly enhanced resistance to *Xoo* and *Xoc* and with agronomic traits similar to those of their parent varieties were obtained by editing the EBEs of the *Xoo*-susceptable genes *OsSWEET11* and *OsSWEET14* as well as the *Xoc*-susceptable gene *OsSULTR3;6* with the CRISPR/Cas9 system. qRT-PCR results revealed that the expression of the susceptibility genes in the edited rice lines cannot be induced anymore by the pathogens harbouring the corresponding TALEs. Further analyses by PCR and sequencing displayed that the obtained edited rice lines are free from transgene and do not contain off-target mutations. This work demonstrates the feasibility of applying the CRISPR/Cas9 system to breed rice varieties resistant to bacterial blight and bacterial leaf streak.

## Supplementary Information

**Additional file 1.**

**Additional file 2.**

**Additional file 3.**

**Additional file 4.**

**Additional file 5.**

## Data Availability

All data generated during this study are included in this published article and its supplementary information files.

## Abbreviations

qRT-PCR: Quantitative reverse transcription polymerase chain reaction
CRISPR: Clustered regularly interspaced short palindromic repeats
Cas9: CRISPR-associated protein 9
