# Supplementary material for: Engineering Resistance to Bacterial Blight and Bacterial Leaf Streak in Rice
Source: Rice (N Y). 2021 Apr 23;14:38. doi: 10.1186/s12284-021-00482-z (PMC8065085; doi:10.1186/s12284-021-00482-z)
Supplement: Supplementary file 1 — Additional file 1. [file 12284_2021_482_MOESM1_ESM.docx]

**Ni et al. Engineering resistance to bacterial blight and bacterial leaf streak in rice**

**Supplemental documents**

**Materials and Methods**:

Bacterial strains and growth conditions

The bacterial strains used in this study are listed in Supplemental Table 1. All *Xanthomonas* strains were grown in nutrient broth (NB, polypeptone 5g/L, beef paste 3g/L, yeast extract 1g/L, sucrose 10g/L) or NB amended with agar at 28℃. Antibiotics were used at the following final concentrations when required: kanamycin 25μg/ml, rifampicin 50μg/ml, and streptomycin 50μg/ml.

Plant materials and inoculations

Rice varieties used in this study include wild type plant: Guihong 1 and Zhonghua 11; mutant plants: GT0105 and ZT0918 (T_4_ and T_5_ generation plants). More information about the rice varieties is available on the China Rice Resource Center (http://www.ricedata.cn). All rice plants were grown under 14-h light (30℃)/10-h dark (28℃) conditions in greenhouses at College of Life Science and Technology, Guangxi University. *Xo* strains were grown overnight, washed once, and resuspended to OD_600_ = 0.5 in sterile distilled water for inoculation to rice. *Xoo* was inoculated into the leaves of 6-week-old rice plants by cutting with scissors previously dipped in the bacterial suspensions (Oliva et al., 2019); *Xoc* was inoculated into the leaves of 6-week-old rice plants by infiltrating with 1ml syringes without needles previously sucked the bacterial suspensions (Zou et al., 2006). Lesion lengths were measured and pictures were taken 14 days post inoculation. The disease assays were performed at least three times.

CRISPR/Cas9-mediated editing of susceptibility genes’ EBEs and detection of off-target effects

Rice varieties were genetically modified with CRISPR/Cas9 technology as described by Ma et al. (2015). SgRNAs were designed with CRISPR MultiTargeter (<http://www.multicrispr.net/index.html>). BLAST searches (http://blast.ncbi.nlm.nih.gov/Blast.cgi) using the target sequences as queries against rice genome sequences were conducted to confirm target specificity. The CRPSPR-P (<http://crispr.hzau.edu.cn/CRISPR2>), and the CRISPR-GE (<http://skl.scau.edu.cn>) were used to predict the potential off-target sites of the designed targets in rice. The PlantCARE (<http://sphinx.rug.ac.be:8080/PlantCARE/>) was used to predict cis-acting regulatory element at the putative off-target sites. The pYLCRISPR vector containing sgRNAs was transferred into Guihong 1 and Zhonghua 11 callus by agrobacterium-mediated transformation (Biorun, Wuhan, China). Genomic DNA was isolated from leaves of transgenic rice using the cetyltrimethylammonium bromide (CTAB) method (Porebski et al., 1997) and used for PCR amplification of the target region with specific primers (Supplemental Table 2). The resulting amplicons were subjected to Sanger sequencing.

RNA isolation and qRT-PCR

The leaves of 6-week-old rice seedlings were infiltrated with different *Xo* strains at OD_600_ = 0.5. At 24 h post inoculation, total RNA was isolated from rice leaves using TRIzol reagent following the manufacturer’s instructions (Invitrogen, #15596018, USA). Total RNA (1 mg) was then used for cDNA synthesis with the RevertAid First Strand cDNA Synthesis Kit (Thermo, #K1622, USA). qRT–PCR was performed using a qTOWER 2.2 Real Time PCR Systems (Analytik Jena, Germany) and ChamQ SYBR Color qPCR Master Mix (Vazyme, Q411-02, China), with the 2^−ΔCt^ method for relative quantification (Livak and Schmittgen, 2001; Soni and Mondal, 2018). The primers used for *OsSWEET11*, *OsSWEET14*, *OsSULTR3;6* and *OsActin1* are listed in Supplemental Table 2. The expression of rice *OsActin1* was used as an internal standard. Each reaction was repeated at least three times.

Evaluation for agronomic traits of the genome editing rice lines

In June 2019, we transplanted 2-week-old rice seedlings into the large pots (Length × width × height of 150cm×100m×25cm. The soil is from rice experimental field of Guangxi University) and the post with seedlings were placed in outdoor and irrigation culture for 150 days until the rice was fully mature. At maturity, the plant height, panicle length, percentage fertility (number of filled grains/total number of grains) and 1000-grain weight were measured.


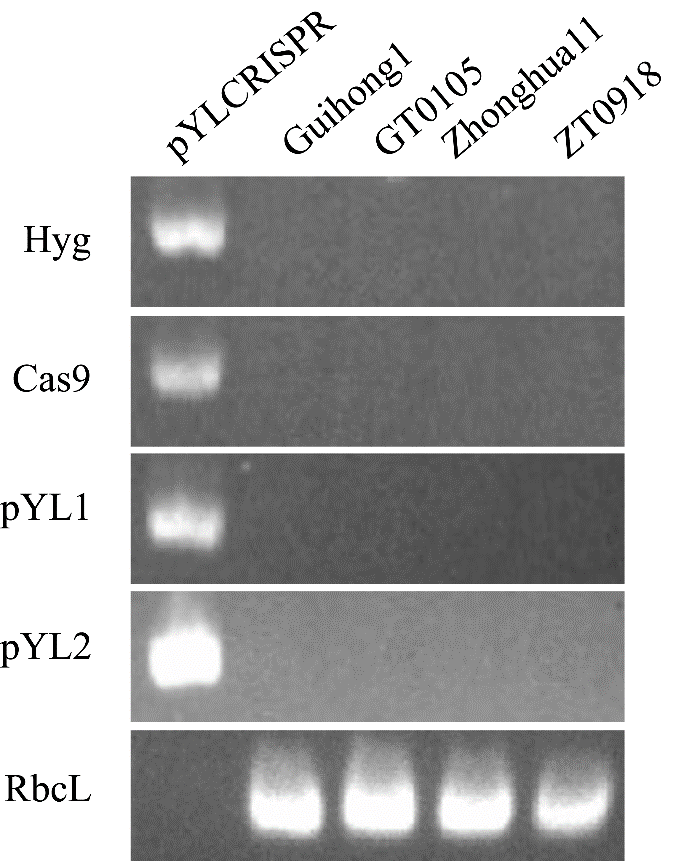


Supplemental Figure 1. Detection of transgenic marker in rice. pYLCRISPR was used as the CRISPR engineering plasmid. Hyg was amplified by internal primers of the *hygromycin* gene; Cas9 was amplified by internal primers of the *cas9* gene; pYL1 and pYL2 was amplified by internal primers of the pYLCRISPR plasmid; RbcL was amplified by internal primers of the rice ribulose-1, 5-bisphosphate carboxylase/oxygenase large subunit gene (*Os01g0791033*), *OsRbcL* was used as an internal control.


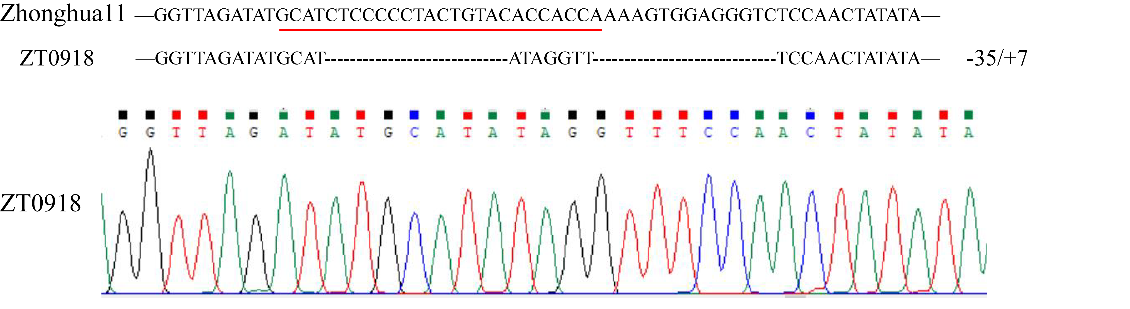


Supplemental Figure 2. Genotypes of *OsSWEET11*’s EBE in ZT0918. The sequences underlined in red are TALE binding sites.

**References:**Livak KJ and Schmittgen TD (2001) Analysis of Relative Gene Expression Data Using Real-Time Quantitative PCR and the 2−ΔΔCT Method. *METHODS* **25**:402-408.

Ma X, Zhang Q, Zhu Q, Liu W, Chen Y, Qiu R, Wang B, Yang Z, Li H, Lin Y, Xie Y, Shen R, Chen S, Wang Z, Chen Y, Guo J, Chen L, Zhao X, Dong Z and Liu Y (2015) A Robust CRISPR/Cas9 System for Convenient, High-Efficiency Multiplex Genome Editing in Monocot and Dicot Plants. *MOL PLANT* **8**:1274-1284.

Oliva R, Ji C, Atienza-Grande G, Huguet-Tapia JC, Perez-Quintero A, Li T, Eom J, Li C, Nguyen H, Liu B, Auguy F, Sciallano C, Luu VT, Dossa GS, Cunnac S, Schmidt SM, Slamet-Loedin IH, Vera Cruz C, Szurek B, Frommer WB, White FF and Yang B (2019) Broad-spectrum resistance to bacterial blight in rice using genome editing. *NAT BIOTECHNOL* **37**:1344-1350.

Porebski S, Bailey LG and Baum BR (1997) Modification of a CTAB DNA extraction protocol for plants containing high polysaccharide and polyphenol components. *PLANT MOL BIOL REP* **15**:8-15.

Soni M and Mondal K K (2018) *Xanthomonas axonopodis* pv. *punicae* uses XopL effector to suppress pomegranate immunity. *Journal of Integrative Plant Biology*, 2018, **60**:341-357.

Zou L, Wang X, Xiang Y, Zhang B, Li Y, Xiao Y, Wang J, Walmsley AR and Chen G (2006) Elucidation of the hrp clusters of Xanthomonas oryzae pv. oryzicola that control the hypersensitive response in nonhost tobacco and pathogenicity in susceptible host rice. *Applied and Environmental Microbiology* **72**:6212-6224.
